# Synthesis and characterization of naphthalimide-functionalized polynorbornenes

**DOI:** 10.1007/s00706-016-1887-3

**Published:** 2016-12-21

**Authors:** Manuel Hollauf, Merima Cajlakovič, Martin Tscherner, Stefan Köstler, Astrid-Caroline Knall, Gregor Trimmel

**Affiliations:** 1 ICTM-Institute for Chemistry and Technology of Materials, NAWI Graz, Graz University of Technology, Stremayrgasse 9, 8010 Graz, Austria; 2 Materials, Institute for Surface Technologies and Photonics, Joanneum Research, Franz-Pichler-Straße 30, 8160 Weiz, Austria

**Keywords:** Ring-opening metathesis polymerization, Polymerization, UV–Vis spectroscopy, Fluorescence, Dyes

## Abstract

**Abstract:**

Highly fluorescent and photostable (2-alkyl)-1*H*-benzo[*de*]isoquinoline-1,3(2*H*)-diones with a polymerizable norbornene scaffold have been synthesized and polymerized using ring-opening metathesis polymerization. The monomers presented herein could be polymerized in a living fashion, using different comonomers and different monomer ratios. All obtained materials showed good film-forming properties and bright fluorescence caused by the incorporated push–pull chromophores. Additionally, one of the monomers containing a methylpiperazine functionality showed protonation-dependent photoinduced electron transfer which opens up interesting applications for logic gates and sensing.

**Graphical abstract:**

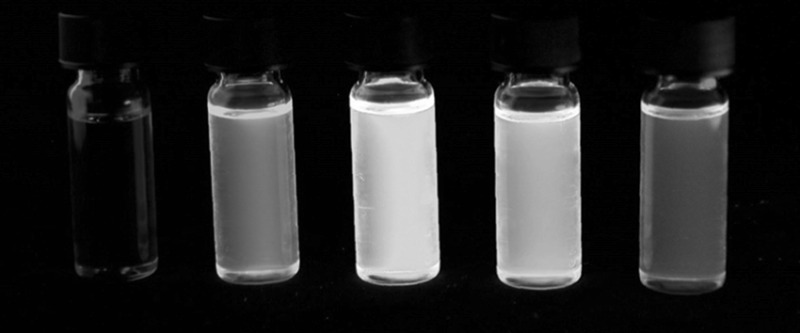

## Introduction

(2-Alkyl)-1*H*-benzo[*de*]isoquinoline-1,3(2*H*)-diones (better known as 1,8-naphthalimides) are highly fluorescent and photostable compounds and are thus in the interest of various fields of technologies. For example, naphthalimides can be used as photoreactive polymerization initiators [1].

Other interesting potential applications of naphthalimide derivatives are related to organic electronics where they are especially applied in organic light emitting diodes (OLEDs) [2, 3] or in organic photovoltaics (in non-fullerene acceptors and *n*-type polymers) [4].

By utilizing readily synthetically available 4-bromonaphthalic anhydride as a starting material, substituents can be straightforwardly introduced [5] yielding push–pull chromophores, whose photophysical properties strongly depend on the substituents in 6-position. These relationships, together with solvent effects and other effects (e.g., *π*–*π* stacking) have been thoroughly studied [5–8].

Furthermore, chemically responsive functionalities which modulate this intramolecular charge transfer can be attached leading to functional fluorophores which can be used for sensors or imaging [9–17]. Furthermore, the photophysical properties can be further tuned by, e.g., connecting a second dye molecule in 6-position as demonstrated in chitosan-based fluorescent materials [18]. Similarly, naphthalimide-based systems were applied as fluorescent markers in molecular biology and imaging applications [19].

Another opportunity to modify naphthalimide-type dyes is to introduce functionalities via the imide nitrogen. This is typically utilized to tune solubility and compatibility or to attach naphthalimides to other materials [18, 20]. Alternatively, polymerizable groups can be attached to obtain functional polymers. One interesting approach is to molecularly imprint these naphthalimide-decorated materials again leading to optochemical probes [21, 22].

All of the above mentioned modification techniques can be combined, leading to photoswitchable, pH-sensitive polymers which were used to detect lysosomes in cancer cells [23].

In many applications, covalent attachment to the polymer matrix controls the distribution of the dye within the material and prevents the dye from leaching out. In particular, for biological labeling or special sensing applications, the dye molecules should be placed at a distinct location of the polymer chain, thus making a rational polymerization and labeling process necessary.

Ring-opening metathesis polymerization (ROMP) [24] has been recognized as a powerful polymerization technique due to its high functional group tolerance. Thus, a number of dye-functionalized polymers have been successfully obtained [25, 26], while the living nature of ROMP allowed the precise placement of dye molecules in dedicated segments of block copolymers and combining them with stimuli-responsive comonomers [27]. This renders ROMP the method of choice for the synthesis of naphthalimide-functionalized polymers which is the objective of this work. Furthermore, the photophysical properties of the obtained materials are characterized and discussed.

## Results and discussion

The overall reaction scheme is depicted in Scheme 1. Starting from 4-bromo-1,8-naphthalic anhydride, imides are prepared which are typically used for tuning the solubility of the dye or connecting the naphthalimide to a material. We use the imide functionality to link the functional naphthalimide chromophores to a norbornene residue which can be polymerized using ring-opening metathesis polymerization (ROMP). In the resulting 4-substituted 1,8-naphthalimide (formally, 6-substituted (2-alkyl)-1*H*-benzo[*de*]isoquinoline-1,3(2*H*)-dione) system, substituents are introduced in 6-position which can be varied and have a strong influence on the photophysical properties [28, 29].

**Figure:**
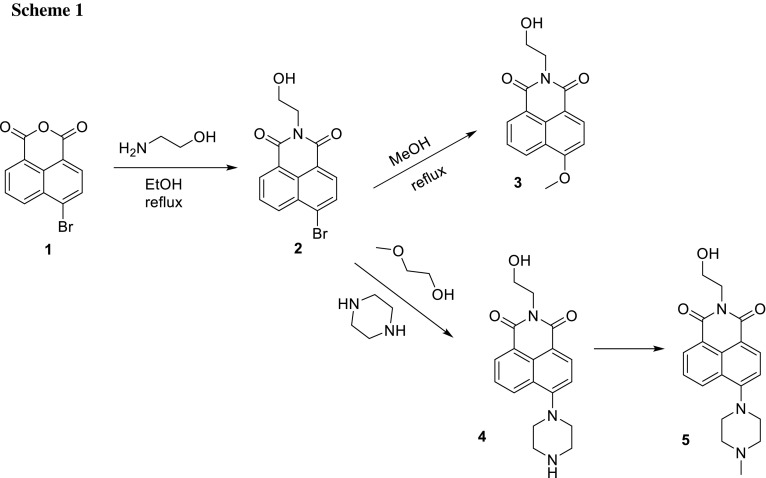

Therefore, we used 4-bromo-1,8-naphthalic anhydride (**1**) as precursor for all synthesized naphthalimide dyes, which is then converted into 6-bromo-2-(2-hydroxyethyl)-1*H*-benzo[*de*]isoquinoline-1,3(2*H*)-dione (**2**), which can be straightforwardly obtained by refluxing **1** with monoethanolamine in ethanol [15].

Due to the fact that bromine is a good leaving group, 2-(2-hydroxyethyl)-6-methoxy-1*H*-benzo[*de*]isoquinoline-1,3(2*H*)-dione (**3**) and 2-(2-hydroxyethyl)-6-(piperazin-1-yl)-1*H*-benzo[*de*]isoquinoline-1,3(2*H*)-dione (**4**) could be obtained using quite mild conditions. **4** was then alkylated using paraldehyde in formic acid [30, 31] which led to 2-(2-hydroxyethyl)-6-(4-methylpiperazin-1-yl)-1*H*-benzo[*de*]isoquinoline-1,3(2*H*)-dione (**5**), see Scheme 1.

Monomers **6** (2-(6-bromo-1,3-dioxo-1*H*-benzo[*de*]isoquinolin-2(3*H*)-yl)ethyl bicyclo[2.2.1]hept-5-ene-2-carboxylate), **7** (2-(6-methoxy-1,3-dioxo-1*H*-benzo[*de*]isoquinolin-2(3*H*)-yl)ethyl bicyclo[2.2.1]hept-5-ene-2-carboxylate), **8** (2-[1,3-dioxo-6-(piperazin-1-yl)-1*H*-benzo[*de*]isoquinolin-2(3*H*)-yl]ethyl bicyclo[2.2.1]hept-5-ene-2-carboxylate), and **9** (2-[6-(4-methylpiperazin-1-yl)-1,3-dioxo-1*H*-benzo[*de*]isoquinolin-2(3*H*)-yl]ethyl bicyclo[2.2.1]hept-5-ene-2-carboxylate)) were obtained by esterification of the hydroxyl moiety of **2**–**5**, respectively, with 5-norbornene-2-carbonyl chloride using Schotten–Baumann conditions (which is shown in Scheme 2). 2-(6-Diethylamino-1,3-dioxo-1*H*-benzo[*de*]isoquinolin-2(3*H*)-yl)ethyl bicyclo[2.2.1]hept-5-ene-2-carboxylate (**10**) was prepared by amination of brominated monomer **6** [32, 33].

**Figure:**
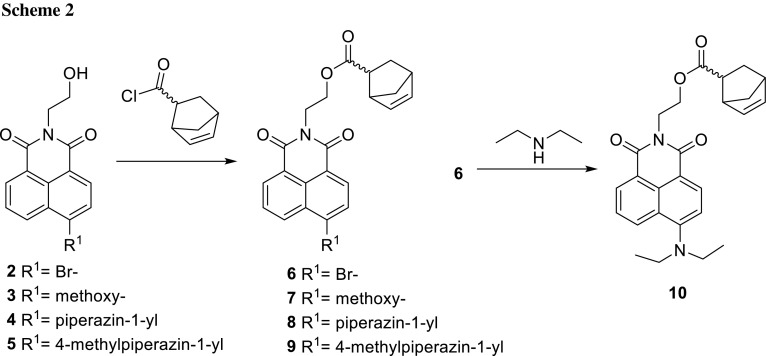

**Fig. 1:**
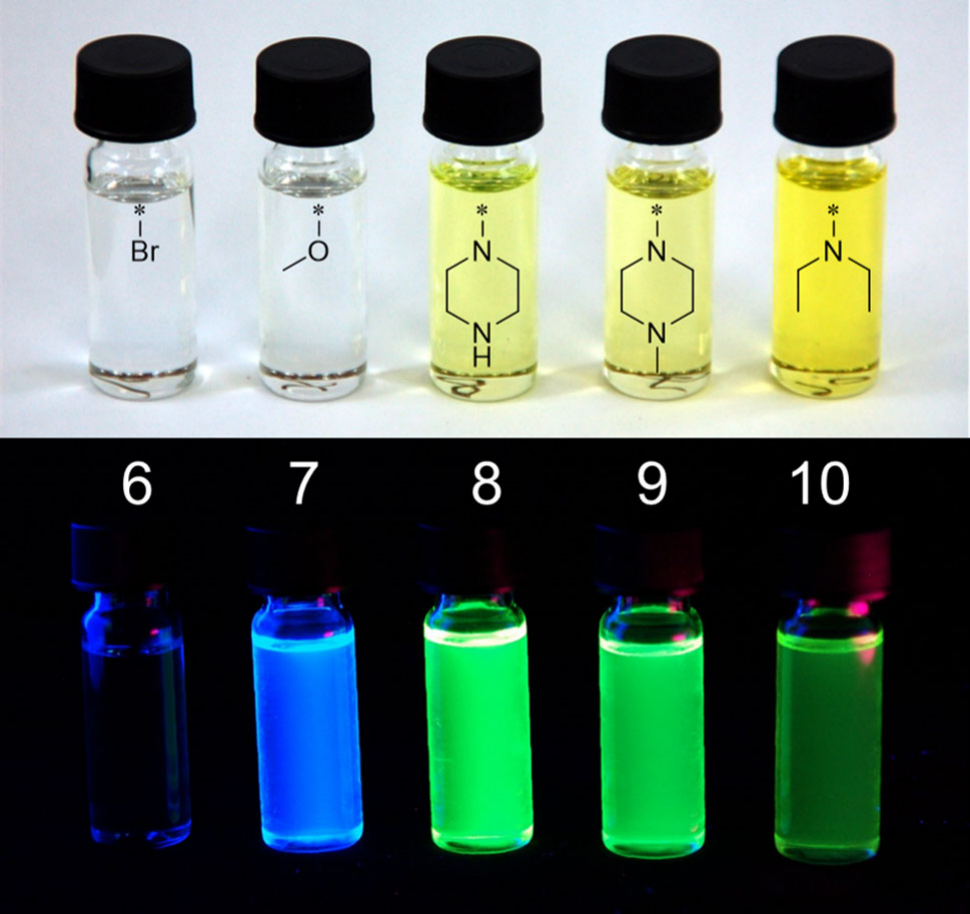
Monomers **6**–**10** in solution (DMSO) under visible light (*top*) and UV irradiation (*λ* = 365 nm)

**Fig. 2:**
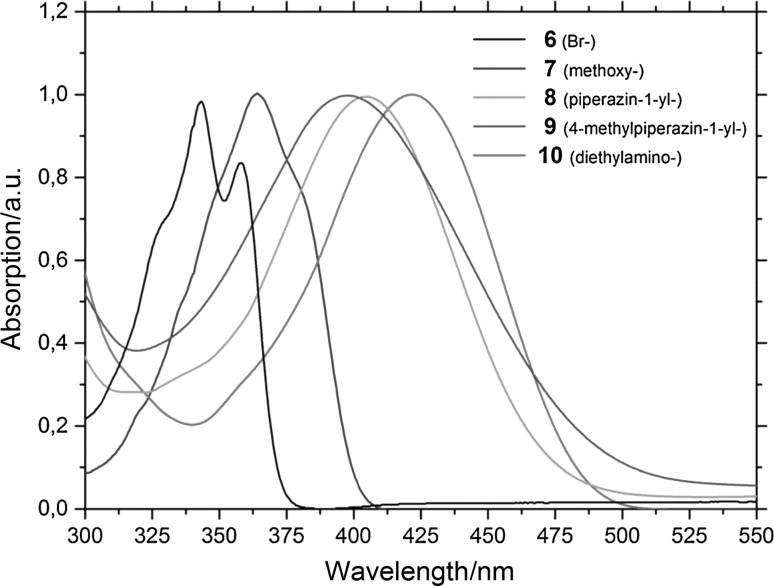
Normalized UV–Vis absorption spectra of monomers **6**–**10** in dichloromethane

As shown in Figs. 1 and 2 as well as in Table 1, the substituent at position 6 in the *N*-alkyl-1*H*-benzo[*de*]isoquinoline-1,3(2*H*)-dione system has a strong influence on the spectroscopic characteristics. Replacing the bromine substituent in **6** with electron-donating substituents creates a donor–acceptor system with the naphthalimide moiety acting as an acceptor, leading to a bathochromic shift. Comparing the absorption maxima of **6**–**10** in DMSO to those in DCM, the absorption maximum of the methoxy substituted derivative **7** is red-shifted in DMSO by about 20 nm compared to the absorption maximum in DCM which is a consequence of the increased solvent polarity. Notably, this effect was not as pronounced for the amino-substituted compounds **8**–**10**. Furthermore, the UV–Vis absorption of naphthalimide-type chromophores is very sensitive to the conformation of the amino moiety which is reflected in **10** having a significantly red-shifted absorption maximum compared to **8** and **9** which expectedly show similar UV–Vis spectra [32].

**Table 1:** Absorption and photoluminescence spectra of naphthalimide-functionalized monomers in different solvents at room temperature and random copolymers **co(M**_**1**_ **-11**_**499**_ **)**, measured in thin film

	$\lambda_{ \hbox{max} }^{\text{abs}} / {\text{nm}}$			*ε*/dm^3^ mol/cm	$\lambda_{ \hbox{max} }^{\text{em}} / {\text{nm}}$	
	CH_2_Cl_2_	DMSO	Polymer thin film	DMSO	CH_2_Cl_2_	Polymer thin film
**6**	343	340	343	21,000	n.d.^a^	n.d.^a^
**7**	363	363	365	13,700	433	424
**8**	405	408	407	9500	505	496
**9**	399	402	397	7400	497	485
**10**	422	428	422	6200	532	509

^a^ **6** is nonfluorescent

Due to the heavy bromine substituent, the fluorescence of **6** is quenched. **7**–**10** show intense fluorescence and the Stokes shifts are between 70 and 100 nm for all compounds. The emission maximum of diethylamino-substituted compound **10** is bathochromically shifted compared to **7**–**9**, **7** being the emitter with the lowest emission wavelength maximum.

Monomers **6**–**10** were copolymerized with dimethyl bicyclo[2.2.1]hept-5-ene-2,3-dicarboxylate (**11**) in different ratios (1/499, 5/495, 10/490, 50/450) using [1,3-bis(2,4,6-trimethylphenyl)-2-imidazolidinylidene]dichloro(3-phenyl-1*H*-inden-1-ylidene)(pyridyl)ruthenium(II) (**M31**) as initiator to yield random copolymers. For the pH-sensitive monomer **9**, additional matrix monomers (bicyclo[2.2.1]hept-5-ene-2,3-diyl)bis(phenylmethanone) (**12**) and 5,6-bis(ethoxymethyl)bicyclo[2.2.1]hept-2-ene (**13**)) were selected for copolymerization. An overview of the used matrix monomers is shown in Scheme 3.

**Figure:**
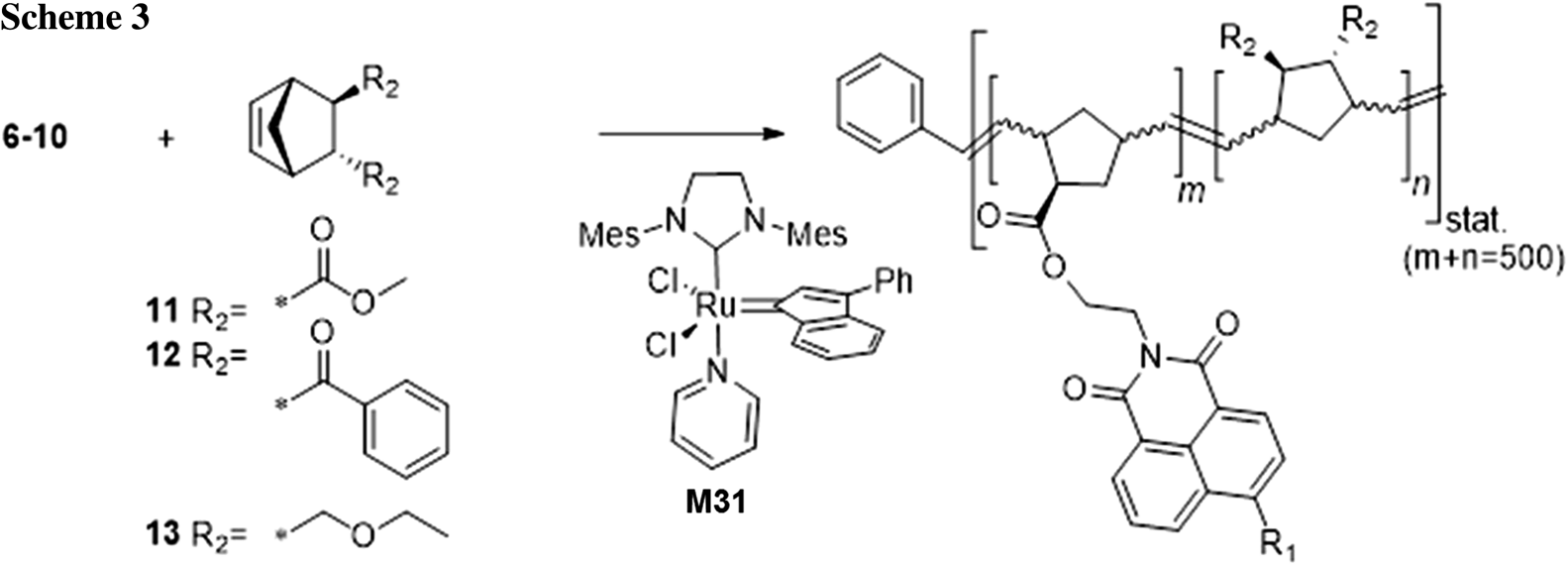

Overall, we observed an increasing polydispersity index (PDI) and shorter chain lengths with increasing dye load. This was especially the case for monomer **8** and **10**; however, dye-functionalized polymeric materials could be successfully prepared using these monomers. Monomer **9** was successfully copolymerized with the two additional comonomers (**12** and **13**, see Scheme 3) as indicated by similar polydispersity indices for all three copolymers. DSC (differential scanning calorimetry) measurements revealed that the glass transition temperature (*T*_g_) was expectedly mainly governed by the bulk comonomers. A small increase in *T*_g_ was detected for monomers **6**–**7** with increasing dye load, whereas increased dye loadings for **8**–**10** led to a decreased *T*_g_.

**Fig. 3:**
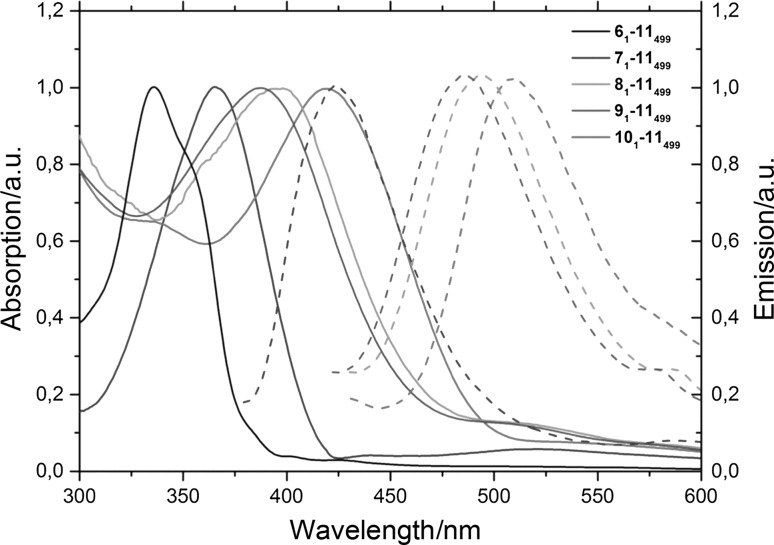
UV–Vis absorption and photoluminescence emission spectra of drop cast polymer films of **co(M**_**1**_ **-11**_**499**_ **)** *M* = **6**,**7**,**8**,**9**, or **10**

UV–Vis absorption and photoluminescence spectra (*λ*_ex_ = 395 nm) of drop-casted films of polymers **co(M**_**1**_ **-11**_**499**_ **)** (*M* = **6**,**7**,**8**,**9**, or **10**) are shown in Fig. 3. Since **6** and its copolymers did not show any fluorescence, no fluorescence data is shown for polymer **co(6**_**1**_ **-11**_**499**_ **)**.

The absorption and photoluminescence of the dye-functionalized monomers and the derived polymers are very similar to the free dye monomers **6**–**10** in solution. Thus, stacking of the naphthalimides which would lead to strong shifts in the photophysical properties is avoided.

**Fig. 4:**
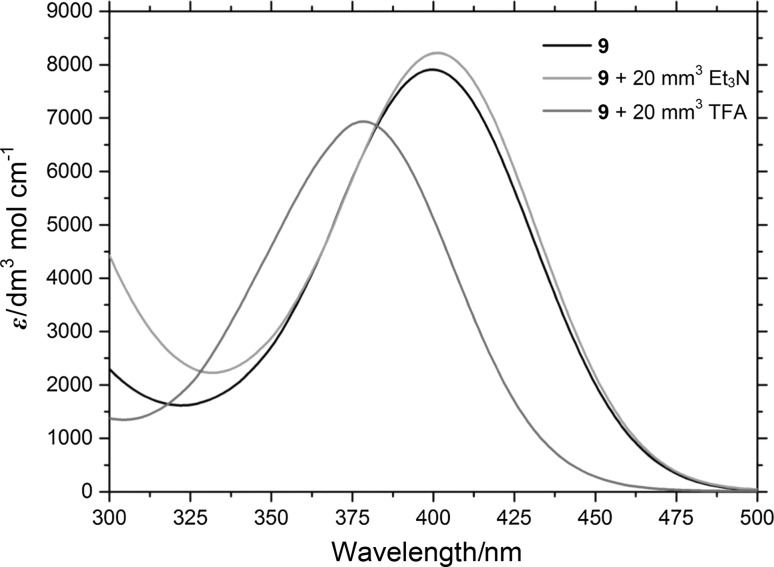
UV–Vis spectra of monomer **9** in DMSO, after addition of trifluoroacetic acid (TFA) and after addition of triethylamine

In addition, monomer **9** and all related copolymers with this monomer are pH-sensitive due to the possibility to protonate the piperazine group. In Fig. 4, the UV–Vis spectra of the protonated and unprotonated form of the monomer are shown. The absorption maximum of **9** at 402 nm is blue shifted to 375 nm if the pyrazine is protonated, e.g., by the addition of trifluoroacetic acid. Upon addition of a base (e.g., triethylamine), the original spectrum is observed so that this reaction can be considered reversible. Thus, copolymers with **9** are possible candidates for polymer based pH-sensing materials, using similar read-out techniques as proposed by Trupp et al. using the same dye functionality in a hydrogel matrix [34]. The possibility to use copolymers of **9** as sensor materials will be exploited in the future.

## Conclusion

In this contribution, different naphthalimide dyes have been successfully linked to norbornene monomers. All derivatives were subsequently copolymerized with different norbornene monomers leading to random copolymers. Narrow polydispersity indices and good control of the molecular weight suggest a living polymerization which can be used to design special macromolecular architectures. The optical properties of the polymers are practically identical to those of the dye monomers showing that *π*–*π* stacking of the naphthalimide dyes is avoided.

## Experimental

Absorption spectra were recorded on a Shimadzu spectrophotometer UV-1800. The emission was measured on a Hitachi F-7000 fluorescence spectrometer equipped with a photomultiplier R928 from Hamamatsu. NMR spectroscopy (^1^H, APT) was done on a Bruker Avance 300 MHz spectrometer. Deuterated solvents (chloroform-d, DMSO-*d*_*6*_) were obtained from Cambridge Isotope Laboratories Inc. Peak shapes are specified as follows: s (singlet), bs (broad singlet), d (doublet), dd (doublet of doublets), t (triplet), q (quadruplet), m (multiplet). Gel permeation chromatography (GPC) was used to determine molecular weights and the polydispersity index (PDI) of the polymers. Measurements were carried out in THF with the following arrangement: a Merck Hitachi L6000 pump, separation columns from Polymer Standards Service (5 µm grade size) and a refractive-index detector form Wyatt Technology. For calibration, polystyrene standards purchased from polymer standard service were used. Glass transition temperatures (*T*_g_) and melting points (m.p.) were measured on a Perkin Elmer Differential Scanning Calorimeter Hyper DSC 8500. Three isothermal cycles were executed, the second scan was analyzed. The scanning speed for cooling and for heating was set to 20 °C/min and the temperature range was set from 20 to 200 °C. MALDI-TOF mass spectrometry was performed on a Micromass TofSpec 2E Time-of-Flight Mass Spectrometer. The instrument is equipped with a nitrogen laser (337 nm wavelength operated at a frequency of 5 Hz) and a time lag focusing unit. Ions were generated by irradiation just above the threshold laser power. Positive ion spectra were recorded in reflectron mode applying an accelerating voltage of 20 kV and externally calibrated with a suitable mixture of poly(ethylene glycol)s (PEG). Analysis of data was done with Mass Lynx-Software V3.5 (Micromass/Waters, Manchester, UK). All chemicals were purchased from commercial sources (Sigma Aldrich, VWR, ABCR) and used as received. Solvents were purified using appropriate drying agents and degassed with nitrogen before use. Catalyst M31 was provided by Umicore, Germany.

### 6-Bromo-2-(2-hydroxyethyl)-1H-benzo[*de*]isoquinoline-1,3(2H)-dione (**2**, C_14_H_10_BrNO_3_)

**1** (2.002 g, 7.226 mmol, 1.0 equiv.) was dissolved in 110 cm^3^ of EtOH in a 250 cm^3^ two-neck round-bottom flask equipped with a stirrer bar. After heating to reflux, then 467 mm^3^ ethanolamine (7.949 mmol, 1.1 equiv.) was added and the reaction mixture was stirred for 4 h. The solution turned from pale beige to dark brown and was cooled to room temperature. The formed precipitate was filtered off, washed three times with dest. H_2_O/EtOH and was dried under vacuum. Yield: 1.804 g (78%) of **2**. ^1^H NMR (300 MHz, DMSO-*d*_*6*_): *δ* = 8.60–8.52 (m, 2H, H_naph_7, H_naph_9), 8.34–8.31 (d, 1H, ^3^ *J*_HH_ = 8.1 Hz, H_naph_4), 8.23–8.20 (d, 1H, ^3^ *J*_HH_ = 8.1 Hz, H_naph_8), 8.02–7.97 (t, ^3^ *J*_HH_ = 7.9 Hz 1H, H_naph_5), 4.83–4.79 (t, 1H, ^3^ *J*_HH_ = 5.7 Hz, OH–CH_2_–), 4.16–4.12 (t, 2H, ^3^ *J*_HH_ = 6.4 Hz, –N–CH_2_–CH_2_–), 3.65–3.59 (m, 2H, OH–CH_2_–CH_2_–) ppm; ^1^H NMR spectra were found to be identical with the ones described in Ref. [15].

### 2-(2-Hydroxyethyl)-6-methoxy-1H-benzo[*de*]isoquinoline-1,3(2H)-dione (**3**, C_15_H_13_NO_4_)

A 50 cm^3^ round-bottom flask equipped with magnetic stir bar and reflux condenser was filled with 207 mg of **2** (0.64 mmol, 1 equiv.) and 45.6 mg potassium hydroxide (0.81 mmol, 1.27 equiv.) and dissolved in 5 cm^3^ MeOH. The reaction mixture was stirred at 70 °C for 4 days. The colorless slurry turned pale yellow. After cooling down to room temperature, 15 cm^3^ H_2_O was added and stored overnight in the fridge. On the next day the yellow precipitate was recovered by filtration and dried in vacuo. Yield: 163.2 mg (94%) of **3**. ^1^H NMR (300 MHz, CDCl_3_): *δ* = 8.62–8.55 (m, 3H, H_naph_4, H_naph_7, H_naph_9), 7.73–7.68 (t, ^3^ *J*_HH_ = 7.9 Hz, 1H, H_naph_8), 7.06–7.04 (d, ^3^ *J*_HH_ = 8.1 Hz, 1H, H_naph_5), 4.46–4.43(t, 2H, ^3^ *J*_HH_ = 5.0 Hz, OH–CH_2_–CH_2_–), 4.00–3.94 (m, 2H, –N–CH_2_–CH_2_–), 2.63–2.60 (t, 1H, ^3^ *J*_HH_ = 5.5 Hz, OH–CH_2_–CH_2_–) ppm; ^1^H NMR spectra were found to be identical with the ones described in Ref. [35].

### 2-(2-Hydroxyethyl)-6-(piperazin-1-yl)-1H-benzo[*de*]isoquinoline-1,3(2H)-dione (**4**, C_18_H_19_N_3_O_3_)

**2** (1 g, 3.12 mmol, 1 equiv.) and 1.015 g piperazine hexahydrate (5.23 mmol, 1.68 equiv.) were dissolved in 20 cm^3^ methoxyethanol, and heated to reflux. After 1 h the reaction mixture started to turn yellow and after continuing the reaction overnight, a yellow solid was formed which was isolated via suction filtration and recrystallized from aqueous ethanol. The crude product was further purified by column chromatography (DCM:MeOH 4/1). After evaporation of the solvent the product was dried in vacuo. Yield: 485 mg (48%) of **4**. ^1^H NMR (300 MHz, DMSO-*d*_*6*_): *δ* = 8.57–8.47 (m, 2H, H_naph_7, H_naph_9), 8.43–8.40 (d, 1H, ^3^ *J*_HH_ = 8.1 Hz, H_naph_4), 7.85–7.81 (t, 1H, H_naph_8), 7.50–7.40 (m, 1H, ^3^ *J*_HH_ = 8.1 Hz, H_naph_5), 4.80 (bs, 1H, –CH–OH), 4.16–4.11 (m, 2H, –N–CH_2_–), 3.62–3.57 (m, 2H, –CH_2_–O–), 3.38 [s, 8H, N–(CH_2_)_2_] ppm; ^1^H NMR spectra were found to be identical with the ones described in Ref. [10]; HRMS (MALDI-TOF): *m/z* = 326.1505 ([M+H]^+^).

### 2-(2-Hydroxyethyl)-6-(4-methylpiperazin-1-yl)-1H-benzo[*de*]isoquinoline-1,3(2H)-dione (**5**, C_19_H_21_N_3_O_3_)

Compound **4** (100 mg, 0.31 mmol, 1 equiv.) and 18.6 mg paraldehyde (0.62 mmol, 2 equiv.) were dissolved in 4 cm^3^ formic acid (88–91%) and stirred at 80 °C overnight. On the next day the solvent was evaporated. The yellow solid was purified via column chromatography (100:10:1 DCM/MeOH/Et_3_N) and dried in vacuo to yield 45 mg (0.13 mmol, 42%) of **5**. ^1^H NMR (300 MHz, CDCl_3_): *δ* = 8.88–8.76 (m, 2H, H_naph_7, H_naph_9), 8.70–8.67 (m, 1H, H_naph_4), 8.02–7.94 (m, 1H, H_naph_8), 7.52–7.47 (t, ^3^ *J*_HH_ = 7.5 Hz, 1H, H_naph_5), 4.23–4.20 (m, 2H, –N–CH_2_–), 4.14 (bs, 1H, –CH_2_–OH), 4.01–3.98 (m, 2H, –CH_2_–OH), 3.53–3.49 [m, 8H, N–(CH_2_)_2_–], 2.57 (s, 3H, CH_3_) ppm; ^1^H NMR spectra were found to be identical with the ones described in Ref. [10].

### 2-(6-Bromo-1,3-dioxo-1H-benzo[*de*]isoquinolin-2(3H)-yl)ethyl bicyclo[2.2.1]hept-5-ene-2-carboxylate (**6**, C_22_H_18_BrNO_4_)

**2** (799 mg, 2.5 mmol) was dissolved in 30 cm^3^ DCM and added dropwise to 1.1 equiv. of norbornyl chloride prepared in situ in 25 cm^3^ dry DCM [from 225 mm^3^ acryloyl chloride and 625 mm^3^ (excess) freshly distilled cyclopentadiene]. Immediately after, 203 mm^3^ (2.5 mmol, 1 equiv.) pyridine and 20 mg (catalytic amount) of DMAP were added. The reaction mixture was stirred overnight. On the next day, the reaction was quenched with 12 cm^3^ distilled water. The organic layer was extracted with HCl (5%), 2% sodium bicarbonate and dried over sodium sulfate. The crude product was concentrated under reduced pressure and purified via column chromatography (100:1 dichloromethane/methanol). Yield 748.5 mg (63%); colorless solid; m.p.: 204 °C; ^1^H NMR (300 MHz, CDCl_3_): *δ* = 8.69–8.66 (d, 2H, ^3^ *J*_HH_ = 8.9 Hz, H_naph_7), 8.61–8.58 (d, 1H, ^3^ *J*_HH_ = 8.9 Hz, H_naph_9), 8.45–8.42 (d, 1H, ^3^ *J*_HH_ = 7.8 Hz, H_naph_4), 8.07–8.05 (d, 1H, ^3^ *J*_HH_ = 7.8 Hz, H_naph_8), 7.89–7.84 (t, 1H, ^3^ *J*_HH_ = 7.3 Hz, H_naph_5), 6.08–6.05 (d, 1H, ^3^ *J*_HH_ = 2.8 Hz, H_nb_6), 5.83–5.80 (d, 1H, ^3^ *J*_HH_ = 2.8 Hz, H_nb_5), 4.40–4.36 (m, 2H, –N–CH_2_–CH_2_–), 4.50–4.46 (m, 2H, –N–CH_2_–CH_2_–), 3.11 (bs, 1H, H_nb_2), 2.91–2.85 (m, 1H, H_nb_1), 2.83 (bs, 1H, H_nb_3^b^), 1.89–1.80 (m, 1H, H_nb_4), 1.37–1.19 (m, 3H, H_nb_7^a,b^, H_nb_3a) ppm (no signals for the *exo*-compound could be detected); ^13^C NMR (75 MHz, CDCl_3_): *δ* = 174.79 (1C, –COO–), 163.70 (2C, O=C–N–C=O), 137.75, 133.55, 132.28, 131.45, 131.28, 130.58, 129.17, 128.25, 123.03, 122.16 (10C, C_naph_), 132.55, 130.80 (2C, C_nb_5, C_nb_6), 61.44 (1C, –COO–CH_2_–CH_2_–), 49.69 (1C, C_nb_7), 45.69 (1C, C_nb_1), 43.33 (1C, C_nb_2), 42.61 (1C, C_nb_4), 39.38 (1C, –N–CH_2_–CH_2_–), 29.41 (1C, C_nb_3) ppm; HRMS (MALDI-TOF): *m/z* = 465.0382 ([M+Na]^+^).

### 2-(6-Methoxy-1,3-dioxo-1H-benzo[*de*]isoquinolin-2(3H)-yl)ethyl bicyclo[2.2.1]hept-5-ene-2-carboxylate (**7**, C_23_H_21_NO_5_)

**3** (1.5 g, 5.53 mmol) was dissolved in 200 cm^3^ DCM and added dropwise to 1.1 equiv. of norbornyl chloride prepared in situ [from 493 mm^3^ acryloyl chloride and 1.37 cm^3^ (excess) freshly distilled cyclopentadiene]. Immediately after the addition 445 mm^3^ (5.53 mmol, 1 equiv.) pyridine and 50 mg (catalytic amount) of DMAP was added. The reaction mixture was stirred overnight. On the next day, the reaction was quenched with 35 cm^3^ distilled water. The organic layer was extracted with HCl (5%), 2% sodium bicarbonate and dried over sodium sulfate. The crude product was concentrated under reduced pressure and purified via column chromatography (5:1 cyclohexane/ethyl acetate). Yield 1.4 g (65%); pale yellow solid; m.p.: 184 °C; ^1^H NMR (300 MHz, CDCl_3_): *δ* = 8.61–8.54 (m, 3H, H_naph_4, H_naph_7, H_naph_9), 7.73–7.67 (t, 1H, ^3^ *J*_HH_ = 8.4 Hz, H_naph_8), 7.05–7.03 (d, 1H, ^3^ *J*_HH_ = 8.4 Hz, H_naph_5), 6.07–6.04 (m, 1H, H_nb_6), 5.83–5.79 (m, 1H, H_nb_5), 4.48–4.35 (m, 4H, –N–CH_2_–CH_2_–), 4.13 (s, 3H, –O–CH_3_), 3.11 (bs, 1H, H_nb_2), 2.93–2.87 (m, 1H, H_nb_1) 2.82 (bs, 1H, H_nb_3^b^), 1.88–1.80 (m, 1H, H_nb_4), 1.38–1.32 (m, 2H, H_nb_3^a^, H_nb_7^a^), 1.21–1.18 (d, ^3^ *J*_HH_ = 8.0 Hz, 1H, H_nb_7^b^) ppm (no signals for the *exo*-compound could be detected); ^13^C NMR (75 MHz, CDCl_3_): *δ* = 174.77 (1C, –COO–), 164.61, 163.98, (2C, O=C–N–C=O), 161.06 137.68, 132.62, 131.80, 128.93, 123.68, 122.36, 115.03, 105.37 (10C, C_naph_), 133.72 (2C, C_nb_5, C_nb_6), 61.60 (1C, –COO–CH_2_–CH_2_–), 56.36 (1C, –O–CH_3_), 49.68 (1C, C_nb_7), 45.71 (1C, C_nb_1), 43.33 (1C, C_nb_2), 42.62 (1C, C_nb_4), 39.00 (1C, –N–CH_2_–CH_2_–), 29.38 (1C, C_nb_3) ppm; HRMS (MALDI-TOF): *m/z* = 414.1042 ([M+Na]^+^).

### 2-[1,3-Dioxo-6-(piperazin-1-yl)-1H-benzo[*de*]isoquinolin-2(3H)-yl]ethyl bicyclo[2.2.1]hept-5-ene-2-carboxylate (**8**, C_26_H_27_N_3_O_4_)

**4** (300 mg, 0.92 mmol) was dissolved in 40 cm^3^ DCM and added dropwise to 1.1 equiv. of norbornyl chloride prepared in situ in 10 cm^3^ dry DCM [from 99 mm^3^ acryloyl chloride and 227 mm^3^ (excess) freshly distilled cyclopentadiene]. Immediately after the addition 75 mm^3^ (0.92 mmol, 1 equiv.) pyridine and 10 mg (catalytic amount) of DMAP was added. The reaction mixture was stirred overnight. After full consumption of the starting material had been detected by TLC (DCM/methanol 20/1), 12 cm^3^ of water were added to quench the reaction which was then stirred for 90 min. The organic layer was extracted with HCl (5%) and 2% sodium bicarbonate, and subsequently dried over sodium sulfate. After solvent removal under reduced pressure, the product was purified using column chromatography (40/1 DCM/MeOH). Yield 269 mg (68%); yellow waxy substance; m.p.: 90 °C (dec.); ^1^H NMR (300 MHz, DMSO-*d*_*6*_): *δ* = 8.62–8.39 (m, 3H, H4, H7, H9), 7.73 (t, 1H, ^3^ *J*_*HH*_ = 7.9 Hz, H8), 7.27–7.17 (m, 1H, H5), 6.11–6.08 (m, 1H, H_nb_6), 5.65–5.62 (m, 1H, H_nb_5) 4.38–4.24 (m, 4H, –N–CH_2_–CH_2_–O–), 3.33 [bs, 8H, N–(CH_2_)_2_–(CH_2_–)_2_], 3.09 (bs, 1H, H_nb_2) 3.00–2.75 (m, 3H, H_nb_1, H_nb_3^b^, H_nb_4), 1.52–1.27 (m, 3H, H_nb_3^a^, H_nb_7^a,b^) ppm [characteristic *exo*-signals: 6.00–5.88 (m, 0.2H)]; ^13^C NMR (75 MHz, DMSO-*d*_*6*_): *δ* = 173.07 (1C, –COO–), 163.84 (2C, O=C–N–C=O), 155.37, 137.58, 136.78, 132.99, 132.54, 130.02, 126.11 (10C, C_naph_), 131.38 (2C, C_nb_5, C_nb_6), 61.48 (1C, –O–CH_2_–CH_2_–), 50.01 (1C, C_nb_7) 45.78 [2C, –N–(CH_2_)–], 45.00 (1C, C_nb_1), 43.21 [2C, –N–(CH_2_)–], 42.89 (1C, C_nb_2), 42.57 (1C, C_nb_4), 39.01 (–N–CH_2_–CH_2_–), 31.05 (1C, C_nb_3) ppm; HRMS (MALDI-TOF): *m/z* = 445.2049 ([M]^+^).

### 2-[6-(4-Methylpiperazin-1-yl)-1,3-dioxo-1H-benzo[*de*]isoquinolin-2(3H)-yl]ethyl bicyclo[2.2.1]hept-5-ene-2-carboxylate (**9**, C_27_H_29_N_3_O_4_)

**5** (300 mg, 0.884 mmol) was dissolved in 40 cm^3^ dry DCM and added dropwise to 10 cm^3^ dry DCM of 1.2 equiv. norbornyl chloride prepared in situ [from 94.3 mm^3^ acryloyl chloride and 219 mm^3^ (excess) freshly distilled cyclopentadiene]. Immediately after, 71.4 mm^3^ (0.884 mmol, 1 equiv.) pyridine and 20 mg (catalytic amount) of DMAP were added. The reaction mixture was stirred overnight. Progress of the reaction was monitored via TLC (DCM:MeOH 20/1). The esterification was completed on the next day and excess acid chloride was quenched with 7 cm^3^ water. The organic layer was then extracted with saturated sodium bicarbonate and dried over sodium sulfate. The drying agent was removed via filtration and the solvent was evaporated to yield a sticky yellow solid which was purified via column chromatography (10/1 DCM/MeOH). Yield 212 mg (52%); yellow waxy substance; m.p.: 90 °C (dec.); ^1^H NMR (300 MHz, DMSO-*d*_*6*_): *δ* = 8.62–8.56 (d, 3H, H4, H7, H9), 8.02–7.97 (t, 1H, ^*3*^ *J*_*HH*_ = 7.8 Hz, H8), 7.53–7.50 (d,^*3*^ *J*_*HH*_ = 7.8 Hz, 1H, H5), 6.17–6.14 (m, 1H, H_nb_6), 5.85–5.81 (m, 1H, H_nb_5), 4.47 (bs, 4H, –N–CH_2_–CH_2_–O–), 3.53 [bs, 4H, N–(CH_2_)_2_–], 3.43 (bs, 3H, –CH_3_) 3.17 (bs, 1H, H_nb_2), 2.96 (bs, 1H, H_nb_1) 2.86–2.80 (m, 2H, H_nb_3^b^, H_nb_4), 2.70 [s, 4H, N–(CH_2_)_2_–], 1.48–1.30 (m, 3H, H_nb_3^a^, H_nb_7^a,b^ CH_3_–) ppm [characteristic *exo*-signals: 6.23–6.19 (m, 0.15H), 5.95–5.91 (m, 0.15H)]; ^13^C NMR (75 MHz, CDCl_3_): *δ* = 173.69 (1C, –COO–), 163.62, 163.03 (2C, O=C–N–C=O), 155.75, 137.43, 129.19, 125.98, 125.25, 115.29, 115.04 (10C, C_naph_), 132.25, 130.65 (2C, C_nb_5, C_nb_6), 60.78 (1C, –COO–CH_2_–CH_2_–), 54.49 (1C, C_nb_7), 54.59 [2C, –N–(CH_2_)–], 52.51 [2C, –N–(CH_2_)–], 48.97 (2C, –N–CH_2_–CH_3_), 45.72 (1C, C_nb_1), 44.95 (1C, C_nb_2), 42.48 (1C, C_nb_4), 41.83 (1C, –N–CH_3_) 28.68(1C, C_nb_3) ppm; HRMS (MALDI-TOF): *m/z* = 460.2236 ([M+H]^+^).

### 2-(6-Diethylamino-1,3-dioxo-1H-benzo[*de*]isoquinolin-2(3H)-yl)ethyl bicyclo[2.2.1]hept-5-ene-2-carboxylate (**10**, C_26_H_28_N_2_O_4_)

**6** (300 mg, 0.96 mmol, 1 equiv.) was placed in a 50 cm^3^ round-bottom flask and dissolved in 10 cm^3^ DMF. After addition of 498 mg diethylamine (6.81 mmol, 10 equiv.) the mixture was stirred overnight. On the next day, the solvent was removed by distillation and the product was purified via column chromatography (10/1 DCM/MeOH). Yield 240 mg (57%); yellow waxy substance; m.p.: 90 °C (dec.); ^1^H NMR (300 MHz, CDCl_3_): *δ* = 8.58–8.56 (d, 2H, H7, H9) 8.50–8.44 (t, 1H, ^3^ *J*_HH_ = 8.5 Hz, H4), 7.68–7.63 (t, 1H, ^3^ *J*_HH_ = 8.0 Hz, H8), 7.22–7.19 (d, 1H, ^3^ *J*_HH_ = 8.0 Hz, H5), 6.07–6.05 (m, 1H, H_nb_6), 5.84–5.81 (m, 1H, H_nb_5), 4.47–4.34 (m, 4H, –N–CH_2_–CH_2_–), 3.45–3.38 (m, 4H, –N–CH_2_–CH_3_), 3.10–3.05 (m, 2H, H_nb_1, H_nb_2), 2.78–2.88 (bs, 1H, H_nb_3^b^), 1.87–1.80 (m, 1H, H_nb_4), 1.52–1.47 (t, 6H, ^3^ *J*_HH_ = 7.4 Hz, –N–CH_2_–CH_3_), 1.36–1.32 (m, 3H, H_nb_3^a^, H_nb_7^a,b^) ppm [characteristic *exo*-signals could not be detected due to peak broadening]; ^13^C NMR (75 MHz, CDCl_3_): *δ* = 174.82 (1C, –COO–), 164.72 (2C, O=C–N–C=O), 137.67, 131.31, 131.21, 125.32, 116.93, (10C, C_naph_), 132,64, 132.33 (2C, C_nb_5, C_nb_6), 61.63 (1C, –COO–CH_2_–CH_2_–), 49.68 (1C, C_nb_7), 47.40 (2C, –N–CH_2_–CH_3_), 45.71 (1C, C_nb_1), 43.33 (1C, C_nb_2), 42.48 (1C, C_nb_4), 38.96 (1C, –N–CH_2_–CH_2_–), 29.39 (1C, C_nb_3), 12.37 (2C, –N–CH_2_–CH_3_) ppm [characteristic *exo*-signals could not be detected due to peak broadening]; HRMS (MALDI-TOF): *m/z* = 432.2079 ([M]^+^).

### General procedure for ring-opening metathesis polymerization: synthesis of **co(6**_**1**_**-11**_**499**_**)**

Matrix monomer **11** (100 mg, 0.48 mmol, 499 equiv.) and 0.42 mg (9.5 × 10^−4^ mmol, 1 equiv.) of naphthalimide monomer **6** were placed in a Schlenk tube and dissolved in 4 cm^3^ of absolute dichloromethane. After degassing, the polymerization was initiated with 1.15 mg of modified second-generation Grubbs initiator RuCl_2_(H_2_IMes)(pyridine)_2_(CHPh) (**M31**, 0.0016 mmol, 1 equiv.). TLC (cyclohexane/ethyl acetate 5/1, KMnO_4_) after 2 h proved full turnover. Subsequently, the polymerization was quenched with 200 mm^3^ of ethyl vinyl ether and stirred for 1 h at room temperature. Afterwards, the volume of the reaction mixture was reduced to 1 cm^3^ and the polymer was precipitated by dropwise addition of this solution to 200 cm^3^ of chilled, vigorously stirred methanol. After having repeated this process twice, the precipitated polymer was collected and dried in vacuo. Copolymerizations using monomers **7**–**10** and matrix monomers **12** and **13** were carried out analogously. Yields, GPC-data, and glass transition temperatures are summarized in Table 2.

**Table 2:** Polymerization data of random copolymers of dye monomers **6**–**10** and matrix monomers **11**–**13**

Polymers	Yield/%	Mn/kDa^a^	PDI^a^	*T*_g_/°C^b^
**co(6**_**1**_ **-11**_**499**_ **)**	85	115.7	1.16	93.6
**co(6**_**5**_ **-11**_**495**_ **)**	82	110.1	1.20	94.3
**co(6**_**10**_ **-11**_**490**_ **)**	80	106.5	1.40	97.5
**co(6**_**50**_ **-11**_**450**_ **)**	78	96.7	1.80	97.8
**co(7**_**1**_ **-11**_**499**_ **)**	84	118.3	1.13	95.3
**co(7**_**5**_ **-11**_**495**_ **)**	83	105.1	1.28	95.9
**co(7**_**10**_ **-11**_**490**_ **)**	80	121.3	1.15	98.6
**co(7**_**50**_ **-11**_**450**_ **)**	82	151.7	1.40	99.1
**co(8**_**1**_ **-11**_**499**_ **)**	75	116.4	1.55	94.8
**co(8**_**5**_ **-11**_**495**_ **)**	68	118.7	3.04	95.3
**co(8**_**10**_ **-11**_**490**_ **)**	65	59.1	2.38	96.1
**co(9**_**1**_ **-11**_**499**_ **)**	75	49.9	1.12	94.4
**co(9**_**5**_ **-11**_**495**_ **)**	76	49.7	1.10	95.5
**co(9**_**10**_ **-11**_**490**_ **)**	74	58.3	1.22	96.2
**co(9**_**5**_ **-12**_**495**_ **)**	80	49.9	1.12	150.1
**co(9**_**5**_ **-13**_**495**_ **)**	78	58.3	1.22	28.3
**co(10**_**1**_ **-11**_**499**_ **)**	73	53.2	1.62	94.4
**co(10**_**5**_ **-11**_**495**_ **)**	75	74.8	1.70	93.5
**co(10**_**10**_ **-11**_**490**_ **)**	70	73.5	2.00	90.9
